# P38/NF-κB/Snail Pathway Is Involved in Caffeic Acid-Induced Inhibition of Cancer Stem Cells-Like Properties and Migratory Capacity in Malignant Human Keratinocyte

**DOI:** 10.1371/journal.pone.0058915

**Published:** 2013-03-13

**Authors:** Ye Yang, Yuan Li, Kebo Wang, Yu Wang, Wenqin Yin, Lei Li

**Affiliations:** 1 Department of Hygiene Analysis and Detection, School of Public Health, Nanjing Medical University, Nanjing, People’s Republic of China; 2 The Key Laboratory of Modern Toxicology, Ministry of Education, Nanjing Medical University, Nanjing, People’s Republic of China; University of Missouri-Columbia, United States Of America

## Abstract

**Background:**

Skin cancer is the most common cancer throughout the world. The epithelial-mesenchymal transition (EMT) and the acquisition of cancer stem cells (CSCs)-like properties emerge as critical steps in the metastasis of human skin cancers. Caffeic acid (CaA) exerts anticarcinogenic effects. However, the effects of CaA on the migratory capability and on the CSCs-like properties of skin cancer cells, and the molecular mechanisms underlying it are not fully understood.

**Methods:**

Malignant HaCaT cells were treated by CaA. Transwell assay was performed to determine that CaA attenuated the migratory capability; Spheroid formation assay was performed to confirm that CaA decreased the CSCs-like phenotype; Treated malignant HaCaT cells were molecularly characterized by RT-PCR, Western blots, Southwestern blot, and immunoprecipitation.

**Results:**

In CaA-treated malignant human keratinocyte (malignant HaCaT cells), inhibition of the migratory capability and CSCs-like phenotype were observed. CaA up-regulated the phosphorylation of p38, and down-regulated the activation of nuclear factor κB (NF-κB)/snail signal pathway. Indeed, p38 decreased the DNA-binding activity of NF-κB to the promoter of *snail* gene, which resulted in the transcriptional inactivation of *snail*. Blockage of p38 attenuated the CaA-induced inhibition of migratory capability and CSCs-like phenotype in malignant HaCaT cells.

**Conclusions:**

CaA attenuates the migratory capability and CSCs-like Properties of malignant human keratinocyte, in which, p38-mediated down-regulation of NF-κB/snail signal pathway is involved.

## Introduction

Skin cancer is the most common cancer throughout the world [1], [2]. For the past two decades, the mortality rate of skin cancer is stable, partly due to the metastatic disease [3]. Treatment options for the metastatic skin cancer continue to evolve on several frontiers. Dacarbazine, currently the only US food and drug administration (FDA) approved chemotherapy for treatment of the metastatic disease, has to date resulted in little or no impact on survival, even when assessed in combination with therapeutics with diverse mechanisms of action [4]. Novel chemotherapeutic agents for the treatment of patients with disseminated malignant skin cancer are urgently needed.

Naturally occurring hydroxycinnamic acid derivatives are reported to have anticancer, anti-inflammatory, and antioxidant properties [5], [6]. Their natural origin and ubiquitous occurrence have prompted strong interest in the use of them as anticancer agents. Caffeic acid (3, 4-dihydroxycinnamic acid, CaA) is the major dietary hydroxycinnamic acid. Recent studies suggest that CaA exerts anticancer effects [7]; CaA exerts protective effects against UVB-induced skin damages by suppressing the activation of interleukin-10 and mitogen-activated protein kinases (MAPKs) in mouse skin [8]; Further, CaA inhibits the activity of Fyn kinase (a key mediator required for UVB-induced skin cancer), which may be involved in the suppression of skin carcinogenesis [9]. However, the effects of CaA on the metastatic capability of skin cancer, and the molecular mechanisms underlying in, are not fully understood.

The epithelial-mesenchymal transition (EMT) is a developmental process by which epithelial cells are converted to mesenchymal cells during embryogenesis, tissue remodelling, and wound healing [10]. During such process, epithelial cells acquire mesenchymal cell properties, and show the reduced intercellular adhesion and the increased invasion [11]. The activation of the EMT program has been implicated as an important step in the metastasis of many human tumors, including skin [10], [12].

A concept recently proposed to explain the characteristics of neoplastic tissues is the existence of self-renewing, stem-like cells within tumors, which have been called ‘cancer stem cells (CSCs)’ [13]. CSCs have been identified in many human cancers, including skin, breast, liver, lung, and so on [1], [12], [14], [15]. Within a tumor, CSCs, which constitute a small portion of neoplastic cells, are defined by their capacity to produce new tumors. For this reason, they have also been termed ‘tumor initiating cells’ [13]. A relationship between the EMT and CSCs has been observed, that during the EMT process, epithelial cells acquire stem cell-like traits and that CSCs exhibit a mesenchymal-like appearance [16]. This link between the EMT process and the induction of CSCs may explain why the EMT program induces tumor initiation and progression.

Here, we found that CaA attenuated the migratory capability and CSCs-like Properties in malignant human keratinocyte (malignant HaCaT cells). CaA improved the phosphorylation of p38, which blocked the activation of nuclear factor κB (NF-κB)/snail signal pathway. Inhibition of p38 abolished the CaA-induced inhibition of migratory capability and CSCs-like phenotype in malignant HaCaT cells.

## Results

### CaA attenuates the migratory capacity of malignant HaCaT cells

**Figure 1:**
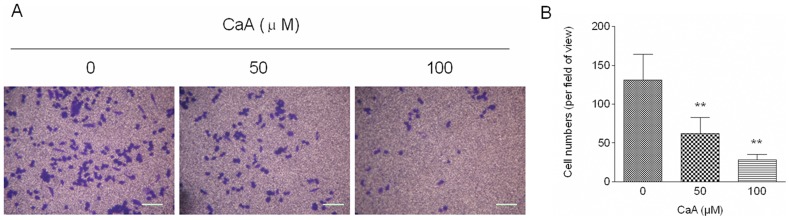
CaA attenuates the migratory capacity of malignant HaCaT cells. Malignant HaCaT cells were treated with 0.0, 50.0, or 100.0 µM of CaA for 48 h, respectively. (A) Transwell assay analyses of the migratory capacity of malignant HaCaT cells, bar  =  125 µm; (B) Quantification of transwell cell migration assay. Migrated cells were stained with crystal violet. The results were expressed as migrated cell numbers per field of view (mean ± SD, n = 5). **p < 0.01 compared with 0.0 µM of CaA-treated malignant HaCaT cells group.

To determine the effects of CaA on the migratory potential of malignant HaCaT cells, transwell assay was performed. As shown in Figure 1, malignant HaCaT cells displayed high migratory capability; in contrast, CaA attenuated the migratory capacity of malignant HaCaT cells in a does-dependent manner. Since 100.0 µM of CaA decreased the migratory capability of malignant HaCaT cells effectively, and since it had no detectable cytotoxicity (Figure S1), we chose this concentration for further investigation.

### CaA induces mesenchymal-epithelial transition (MET) in malignant HaCaT cells

**Figure 2:**
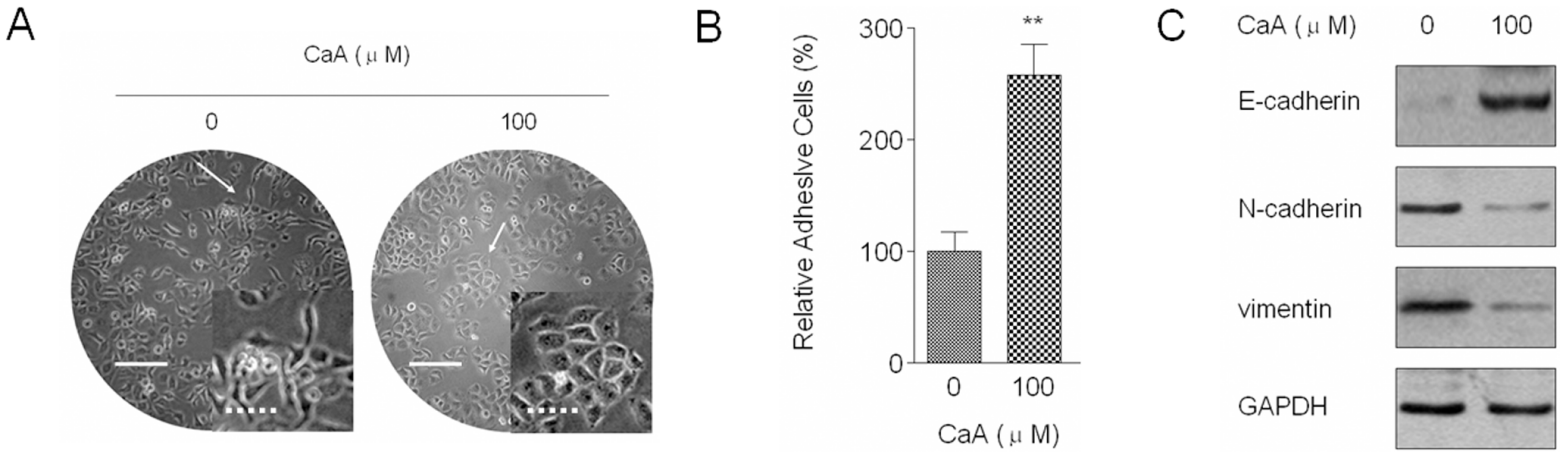
CaA induces MET in malignant HaCaT cells. Malignant HaCaT cells were treated with 0.0 or 100.0 µM of CaA for 48 h, respectively. (A) Morphological images of malignant HaCaT cells (bars: solid line  =  500 µm and dotted line  =  125 µm); (B) Quantification of adhesion assay as described in the section *Materials and Methods*. We used the adhesion ratios of non-treated malignant HaCaT cells to determine the 100% level; (C) Western blot analyses of E-cadherin, N-cadherin, and vimentin levels. Blots were normalized by use of GAPDH to correct for differences in loading of the proteins. **p < 0.01 compared with 0.0 µM of CaA-treated malignant HaCaT cells group.

For the malignant HaCaT cells, alteration from epithelial to spindle-like mesenchymal morphology is a manifestation [17]. Since EMT enables cell to move and invade [18], we then determined the effects of CaA on the EMT process in malignant HaCaT cells. As shown in Figure 2A, malignant HaCaT cells displayed a fibroblast-like mesenchymal appearance; however, after these cells were exposed to CaA for 48 h, they showed an epithelial-like morphology. Inhibition of cellular adhesive ability is associated with EMT initiation [18]. Here, adhesion assays showed that CaA improved the adhesive ability of malignant HaCaT cells (Figure 2B). Then the effects of CaA on the expression of EMT/adhesive markers: E-cadherin, N-cadherin, and vimentin, were determined. After malignant HaCaT cells were treated by CaA for 48 h, E-cadherin level was increased, in contrast, N-cadherin and vimentin levels were decreased (Figure 2C). Hence, both morphological and molecular changes demonstrate that, with exposure to CaA, malignant HaCaT cells undergo a MET.

### CaA decreases the CSCs-like properties of malignant HaCaT cells

**Figure 3:**
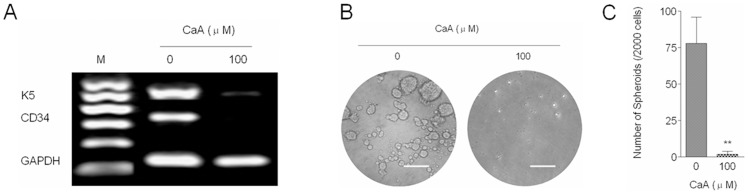
CaA decreases the CSCs-like properties of malignant HaCaT cells. Malignant HaCaT cells were treated with 0.0 or 100.0 µM of CaA for 48 h, respectively. (A) RT-PCR analyses of *K5* and *CD34* mRNA levels. Bands were normalized by use of GAPDH to correct for differences in loading of the cDNAs; (B) Free-floating, viable spheres formed by malignant HaCaT cells (bar  =  125 µm); (C) Sphere quantitation (mean ± SD, n = 3). **p < 0.01 compared with 0.0 µM of CaA-treated malignant HaCaT cells group.

Induction of EMT has been associated with the acquisition of stem cell-like features, including the expression of such stem cells-surface markers, nonadherent growth, and changes in expression of cell-surface glycoproteins [16]. CD34 and K5 are cell-surface markers of skin stem cells [19], [20]. In our present study, malignant HaCaT cells showed elevated expression of *CD34* and *K5* mRNAs; however, after treatment of malignant HaCaT cells with CaA for 48 h, a decreased expression of such mRNAs was observed (Figure 3A). Formation of spheroids demonstrates the capacity of cells for self-renewal and for initiation of tumors, which are characteristics of cancer stem cells (CSCs) [21]. We then determined the effects of CaA on the formation of spheroids in malignant HaCaT cells. In nonadherent dishes, malignant HaCaT cells formed free-floating, viable spheres; however, after treatment of malignant HaCaT cells with CaA for 48 h, such phenomenon was disappeared (Figure 3B and 3C). These data demonstrate that CaA decreases the CSCs-like properties of malignant HaCaT cells.

### CaA inhibits the activation of NF-κB/snail signal pathway by p38

**Figure 4:**
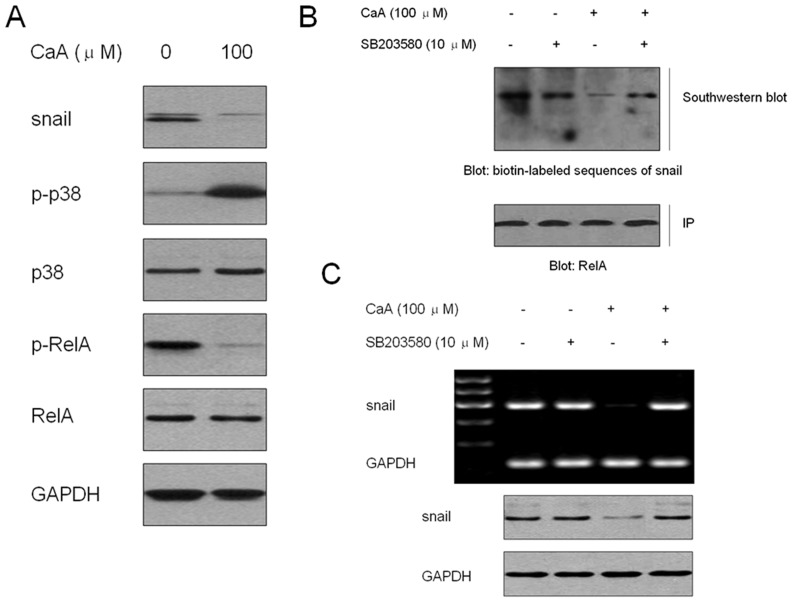
CaA blocks the NF-κB/snail signal pathway by p38. (A) Malignant HaCaT cells were treated with 0.0 or 100.0 µM of CaA for 24 h, respectively. Western blot analyses of snail, p-p38, and p-RelA levels. Blots were normalized by use of GAPDH to correct for differences in loading of the proteins; (B) After malignant HaCaT cells were pretreated by 10.0 µM of SB203580 (a p38 inhibitor) for 6 h, they were exposed to 0.0 or 100.0 µM of CaA for 24 h, respectively. (B, top) Cells lysates were subjected to immunoprecipitation with NF-κB/RelA antibody, and the immunoprecipitates were subjected to Southwestern blots to determine the binding of NF-κB to DNA sequences of *snail* promoter (5’-*taa*-*ggg-agt-tgg-cgg*-3’); (B, bottom) the immunoprecipitates were subjected to Western blot to determine the RelA level, which was used to correct for differences in loading of the immunoprecipitates; (C) malignant HaCaT cells were treated as described in (B), RT-PCR (top) and Western blot (bottom) analyses of snail mRNA and protein levels. Blots/ bands were normalized by use of GAPDH to correct for differences in loading of the proteins/ cDNAs.

Snail, a zinc finger transcriptional factor, functions as a regulator to suppress the expression of adhesion molecules and to assist the escape of tumor cells from cell death during EMT [22]. NF-κB, a key mediator involved in the malignant transformation of HaCaT cells [17], up-regulates snail expression and induces EMT [23], [24]. We hypothesized that NF-κB/snail signal pathway might be involved in the CaA-induced inhibition of CSCs-like properties and migratory capacity in malignant HaCaT cells. Here, as shown in Figure 4A, CaA decreased the expression of phospho-RelA (indicating the activation of NF-κB) and snail, which suggested that CaA blocked the activation of NF-κB/snail signal pathway.

To further determine the up-stream regulator of NF-κB/snail in CaA treated malignant HaCaT cells, we investigated the activation of p38 (an inhibitor of NF-κB and EMT [25]). As shown in Figure 4A, CaA improved the phosphorylation of p38 (indicating the activation of p38). Based on these data, we hypothesized that in malignant HaCaT cells, CaA blocked the NF-κB/snail signal pathway by p38.

We then used immunoprecipitation and Southwestern blot assay to confirm our hypothesis. SB203580 is a specific inhibitor of p38, and we used 10.0 µM of SB203580 to block the CaA-induced activation of p38 (Figure S2). After malignant HaCaT cells were pretreated with 10.0 µM of SB203580 for 6 h, they were exposed to 0.0 or 100.0 µM of CaA for 24 h, respectively. RelA was immunoprecipitated with its specific antibody, and the immunoprecipitates were then subjected to Southwestern blots using the biotin-labeled probe “*gggagttggc*” (Figure S3) to determine the binding of NF-κB to DNA sequences of *snail* promotor. As shown in Figure 4B, CaA decreased the binding of NF-κB to *snail* promotor in malignant HaCaT cells; however, inhibition of p38 abolished this effect. Further, blockage of p38 attenuated the CaA-mediated decreased expression of snail mRNA and protein levels (Figure 4C). These results suggest that, in malignant HaCaT cells exposed to CaA, p38 decrease the DNA-binding activity of NF-κB to *snail* promotor, which results in the transcriptional down-regulation of snail.

### P38 is involved in the CaA-induced MET of malignant HaCaT cells

**Figure 5:**
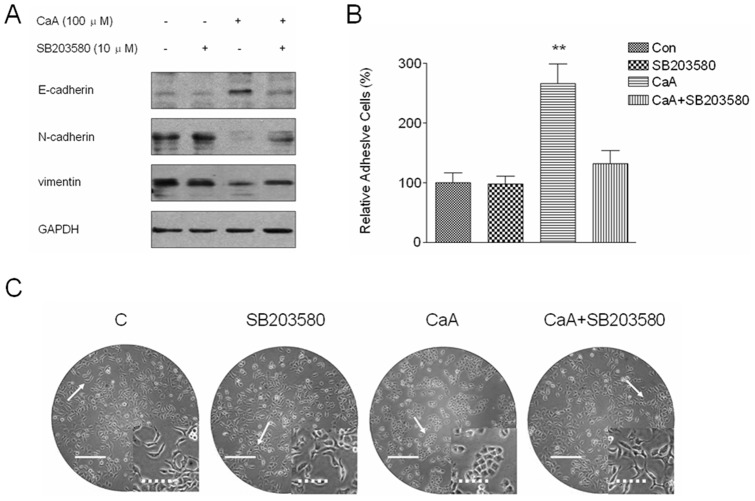
CaA induces MET in malignant HaCaT cells by p38. After malignant HaCaT cells were pretreated by SB203580 (a p38 inhibitor) for 6 h, they were exposed to 0.0 or 100.0 µM of CaA for 48 h, respectively. (A) Western blot analyses of E-cadherin, N-cadherin, and vimentin levels. Blots were normalized by use of GAPDH to correct for differences in loading of the proteins; (B) Quantification of adhesion assay as described in the section *Materials and Methods*. We used the adhesion ratios of non-treated malignant HaCaT cells to determine the 100% level; (C) Morphological images of malignant HaCaT cells (bars: solid line  =  500 µm and dotted line  =  125 µm). **p < 0.01 compared with (CaA + SB203580)-treated malignant HaCaT cells group.

We then determined the functions of p38 in CaA-mediated MET in malignant HaCaT cells. After malignant HaCaT cells were pretreated with 10.0 µM of SB203580 for 6 h, they were exposed to 0.0 or 100.0 µM of CaA for 48 h, respectively. As shown in Figure 5. In CaA-treated malignant HaCaT cells, E-cadherin level was increased, but N-cadherin and vimentin levels were decreased (Figure 5A); cellular adhesive ability was improved (Figure 5B); and cells acquired an epithelial-like morphology (Figure 5C). However, inhibition of p38 abolished the phenomenon above induced by CaA (Figure 5A, 5B, and 5C). These results suggest that p38 is involved in CaA-induced MET of malignant HaCaT cells.

### P38 is involved in CaA-induced inhibition of CSCs-like properties in malignant HaCaT cells

**Figure 6:**
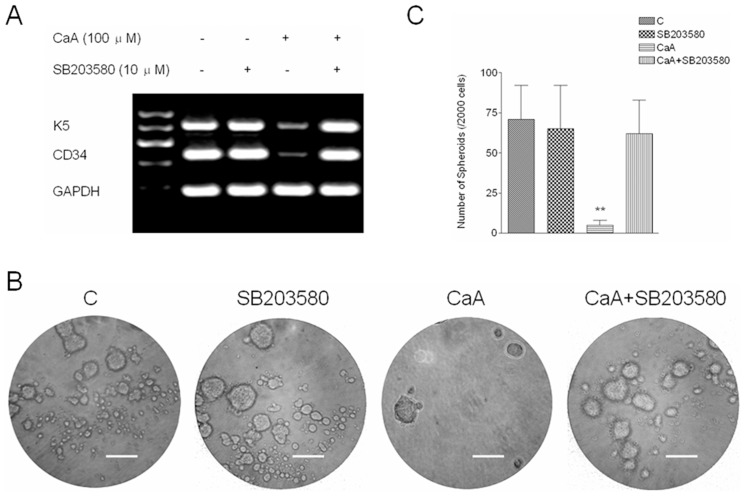
CaA decreases the CSCs-like properties of malignant HaCaT cells by p38. After malignant HaCaT cells were pretreated by SB203580 for 6 h, they were exposed to 0.0 or 100.0 µM of CaA for 48 h, respectively. (A) RT-PCR analyses of *K5* and *CD34* mRNA levels. Bands were normalized by use of GAPDH to correct for differences in loading of the cDNAs; (B) Free-floating, viable spheres formed by malignant HaCaT cells (bar  =  125 µm); (C) Sphere quantitation (mean ± SD, n = 3). **p < 0.01 compared with (CaA + SB203580)-treated malignant HaCaT cells group.

Further, we determined the effects of p38 on CaA-induced inhibition of CSCs-like properties in malignant HaCaT cells. Cells were treated as described above. As shown in Figure 6, CaA attenuated the expression of *CD34* and *K5* mRNAs (Figure 6A), and decreased the formation of spheroids (Figure 6B and 6C). However, inhibition of p38 abolished the phenomenon above induced by CaA (Figure 6A, 6B, and 6C). These results indicate that p38 is involved in CaA-induced inhibition of CSCs-like properties in malignant HaCaT cells.

### CaA attenuates the migratory capacity of malignant HaCaT cells by p38

**Figure 7:**
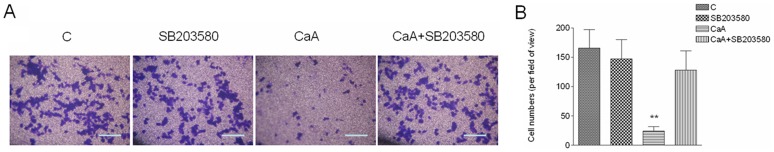
CaA attenuates the migratory capacity of malignant HaCaT cells by p38. After malignant HaCaT cells were pretreated by SB203580 for 6 h, they were exposed to 0.0 or 100.0 µM of CaA for 48 h, respectively. (A) Transwell assay analyses of the migratory capacity of malignant HaCaT cells, bar  =  125 µm; (B) Quantification of transwell cell migration assay. Migrated cells were stained with crystal violet. The results were expressed as migrated cell numbers per field of view (mean ± SD, n = 5). **p < 0.01 compared with (CaA + SB203580)-treated malignant HaCaT cells group.

Finally, we determined if CaA decreased the migratory capacity of malignant HaCaT cells via p38. Malignant HaCaT cells were exposed to CaA (0.0 or 100.0 µM) in the presence or absence of SB203580 as described in Figure 6, and the migratory potential of malignant HaCaT cells was determined by transwell assay. As shown in Figure 7, CaA attenuated the migratory capacity of malignant HaCaT cells; however, inhibition of p38 abolished this phenomenon. These data suggest that p38 is involved in CaA-induced inhibition of migratory capacity in malignant HaCaT cells.

## Discussion

CaA is one of the major metabolites produced by the hydrolyzation of chlorogenic acid, a major phenolic phytochemical in various foods, including coffee [8]. Because a large amount of chlorogenic acid is absorbed in the metabolized form, considerable attention has been focused on the biological effects of metabolites such as CaA in order to evaluate possible *in vivo* effects of chlorogenic acid-containing diets [9]. Accumulating evidence suggests that CaA has the potential to inhibit skin cancer development, for example, CaA inhibits skin tumor promotion induced by 12-O-tetradecanoylphorbol-13-acetate in mouse skin [26]. However, the effects of CaA on the migratory capability of malignant skin cells, and the molecular mechanisms underlying in, remain unclear. Here we found that CaA attenuated the migratory capability and CSCs-like Properties of malignant human keratinocyte, in which, p38-mediated down-regulation of NF-κB/snail signal pathway was involved.

EMT refers to a program during normal embryonic development featuring a loss of epithelial properties, such as cell adhesion and expression of the epithelial marker, E-cadherin, and acquisition of mesenchymal properties, such as increased cell motility and expression of the mesenchymal marker, N-cadherin and vimentin [10]. EMT is viewed as an important step in tumor invasion and metastasis [27]. Here we found that, after exposure of malignant HaCaT cells to CaA for 48 h, they showed an epithelial-like morphology and an improved adhesive ability. Further, there were increased expression of E-cadherin (an epithelial marker), and decreased expression of N-cadherin and vimentin (mesenchymal markers). These results demonstrate that, with exposure to CaA, malignant HaCaT cells undergo a MET.

Acquisition of CSCs-like properties emerge as a critical step in the cancer progression [28]. Many genes are expressed in skin CSCs, including *CD34*, *K5*, and so on [19], [20]. In skin, *CD34* is specifically expressed in keratinocyte SCs, and *CD34* expression is key to skin carcinogenesis [19]; *K5* is a marker of undifferentiated skin SCs or CSCs [20]. In our present study, expressions of *CD34* and *K5* mRNA were substantially decreased in the CaA-treated malignant HaCaT cells. SCs and CSCs share a variety of properties, including selfrenewal, although it is typically dysregulated in oncogenesis. Many cultured SC or CSC lines form free-floating spherical clusters of viable cells containing a preponderance of the SCs or CSCs [21], [29]. In the present study, CaA-treated malignant HaCaT cells exhibited attenuated capacity for forming spheres. These results indicate that malignant HaCaT cells lost CSCs-like characteristics by exposure to CaA.

Snail, a zinc finger transcriptional factor, functions as a regulator to suppress the expression of adhesion molecules and to assist the escape of tumor cells from cell death during EMT [22], [30]. Snail is frequently expressed in many types of tumor cells in which E-cadherin expression is reduced. Moreover, this inverse relationship of snail and E-cadherin was often observed in the invasive types of tumors [31]. Later studies confirmed a suppressing effect of snail on *E-cadherin* promoter via a specific binding to the three E-boxes *(cacctg)* in the promoter region at -178 to +92 of the *E-cadherin* gene [31]. Moreover, snail also has been linked to the acquisition of CSC-like characteristics [22]. Here in CaA-treated malignant HaCaT cells, a decreased expression of snail was observed.

To further determine the up-stream regulator of snail in CaA treated malignant HaCaT cells, we investigated the activation of NF-κB. NF-κB is thought to initiate and accelerate tumorigenesis, and its inhibition blocks cell transformation induced by tumor promoters [16], [23]; NF-κB induces morphological changes, cell migration, snail activation and repression of E-cadherin production [24], [32]. NF-κB binds to *snail* promoter and transcriptional upregulates snail expression [23]. Here, in CaA-treated malignant HaCaT cells, a decreased expression of p-RelA was observed. Further, CaA down-regulated the DNA-binding activity of NF-κB to *snail* promotor, which resulted in the transcriptional inhibition of snail. These results indicate that CaA induces an inactivation of NF-κB/snail signal pathway.

**Figure 8:**
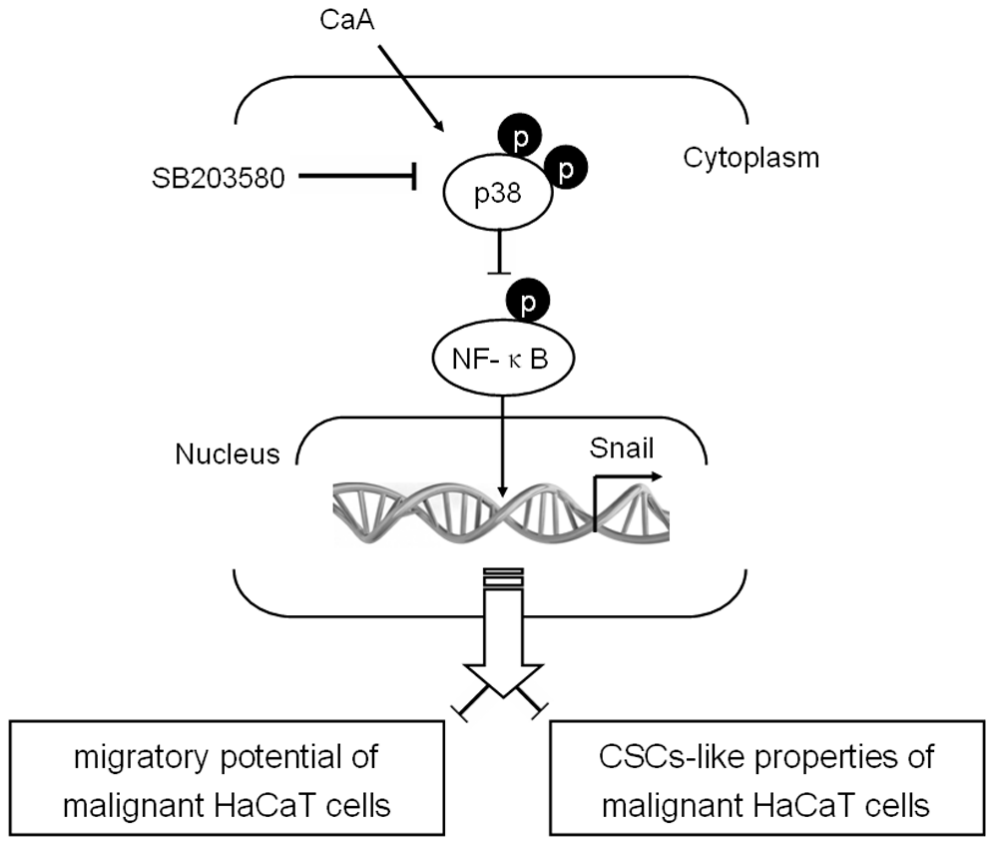
P38/NF-κB/Snail pathway is involved in CaA-induced inhibition of CSCs-like properties and migratory capacity in malignant human keratinocyte. In malignant HaCaT cells exposed to CaA, p38 decreases the DNA-binding activity of NF-κB to the *snail* promotor, which results in the transcriptional down-regulation of snail. Such molecular process causes the inhibition of CSCs-like properties and migratory capacity of malignant HaCaT cells.

The MAPK pathways transduce signals that lead to diverse cellular responses such as cell growth, differentiation, proliferation, apoptosis, and stress responses to environmental stimuli [33]. The extracellular signal regulated kinase 1/2 (ERK1/2) pathway typically transduces growth factor signals that lead to cell differentiation or proliferation, whereas cytokines and stress signals activate the c-Jun N-terminal kinases (JNKs) and p38 MAPK pathways, resulting in stress responses, growth arrest, or apoptosis [34]. Besides being a central mediator of the inflammatory and stress response, p38 plays an important role in non-inflammatory processes such as cell-cycle regulation and cell differentiation [35]. Once activated, p38 phosphorylates a wide array of substrates in the cytoplasm and in the nucleus, thus regulating gene expression, cell cycle and cellular polarization. Studies into the roles of MAPK families in the genesis of EMT have produced conflicting results, due to the heterogeneity of the cellular models and the different experimental approaches used [25], [36]. Previous study demonstrates that p38 promotes E-cadherin expression by suppressing TGF-β-activated kinase 1 (TAK1)-NF-κB signaling [25]. Here, in CaA-treated malignant HaCaT cells, p38 decreases the DNA-binding activity of NF-κB to *snail* promotor, which results in the transcriptional down-regulation of snail. The p38/NF-κB/Snail pathway is involved in CaA-induced inhibition of CSCs-like properties and migratory capacity in malignant human keratinocyte (Figure 8).

## Materials and Methods

### Cell culture and reagents

The malignant transformed HaCaT cells were previously developed by our laboratory [17]. Cells were maintained in 5% CO_2_ at 37°C in RPMI-1640 medium (Life Technologies/Gibco, Grand Island, NY) supplemented with 10% fetal bovine serum (FBS, Life Technologies/Gibco), 100 U/ml penicillin, and 100 µg/ml streptomycin (Life Technologies/Gibco, Gaithersburg, MD). The p38 inhibitor, SB203580 was purchased from Cell Signaling Technology (Beverly, MA, USA). All other reagents used were of analytical grade or the highest grade available.

### Transwell assay

Transwell assay was performed by using of growth factor-reduced matrigel-coated (8 µm pore size, BD, Franklin Lakes, NJ) filters in 24-well plates. Briefly, cells were trypsinized and seeded onto the upper chamber of the transwells (3 × 10^4^ cells/well) in supplements-free 1640 medium. The lower chambers of the transwells were filled with the 1640 medium containing 100 ng/ml of epidermal growth factor (EGF, R&D Systems). The chambers were incubated at 37°C with 5% CO_2_ for 24 h. At the end of incubation, cells on the upper surface of the filter were removed using a cotton swab. Cells migrating through the filter to the lower surface were fixed with 4% paraformaldehyde for 10 minutes and stained with 0.1% crystal violet for 5 minutes. Migrated cells were viewed and photographed under a phase-contrast microscope (Olympus, Tokyo, Japan), and counted in five randomly chosen fields.

### Adhesion assay

A total of 1 × 10^4^ treated cells were seeded in a 96-well plate for 24 h, followed by washing in phosphate-buffered saline (PBS) twice and fixing with 70% ethanol for 15 min at room temperature. The remaining adherent cells were evaluated by WST-8 hydrolysis using Cell Counting Kit-8 (CCK-8, Dojindo Molecular Technologies, Inc.). Briefly, after cells were washed in PBS, they were incubated with 20.0 µl of CCK-8 solution for 4 h. The absorbance at 450 nm was measured with a multi-well plate reader (Model 680, Bio-Rad, USA). The ratios of non-treated control cells were used to determine the 100% level.

### Western blots

Cell lysates were separated by sodium dodecyl sulfate-polyacrylamide gel electrophoresis and transferred to polyvinylidene fluoride membranes (Millipore, Billerica, MA, USA); the immune complexes were detected by enhanced chemiluminescence (Cell Signaling Technology). Antibodies used were E-cadherin, N-cadherin, vimentin, snail, p38, p-p38 (Thr180/Tyr182), RelA, p-RelA (Ser536, Cell Signaling Technology); Glyceraldehyde 3-phosphate dehydrogenase (GAPDH, Sigma, St. Louis, MO). Blots were normalized by use of GAPDH to correct for differences in loading of the proteins.

### Reverse-transcriptase polymerase chain reaction (RT-PCR)

Total RNA (2 µg) was transcribed into cDNA using AMV Reverse Transcriptase (Promega, Madison, WI, USA). Primers for *CD34* (forward, 5′-TTGGGCATCACTGGCTATTT-3′; reverse, 5′-GGAAGGGTTGGGCGTAAGA-3′), *K5* (forward, 5′-GAGCAGGGCACCAAGAC-3′; reverse, 5′-CTCCGCATCAAAGAACATC-3′), and *snail* (forward, 5′- TTCTCCCGAATGTCCCT -3′; reverse, 5′-TCAGCCTTTGTCCTGTAGC -3′) were used for PCR amplification. The PCR reaction was evaluated by checking the PCR products on 2% w/v agarose gels. Bands were normalized by use of GAPDH to correct for differences in loading of the cDNAs

### Spheroid formation

In nonadherent dishes (Costar, US), Cells (1 × 10^4^) were suspended in defined, serum-free medium composed of DMEM/F-12 (Gibco), 10 ng/ml of human recombinant basic fibroblast growth factor (bFGF, R&D Systems, USA), and 10 ng/ml of EGF. Cells were grown for 10 days and fed every 48 h. Total spheres were then counted under a microscope (Olympus).

### Immunoprecipitation

Cells were extracted for 30 min with lysis buffer. After centrifugation of the preparations, the supernatants were incubated with RelA antibodies and subsequently with A+G Sepharose beads (Sigma) at 4°C overnight. The pellets were washed three times, re-suspended in the sodium dodecyl sulfate sample buffer, and boiled to remove protein from the beads. The immunoprecipitates were analyzed by Southwestern blots with a biotin-labeled probe (5’-*taa*-*ggg-agt-tgg-cgg*-3’), or by Western blots.

### Southwestern assays

Southwestern analyses were performed as described previously [17]. Briefly, the immunoprecipitates were separated by SDS–PAGE and transferred to nitrocellulose membranes (Millipore). After the transfer, the filters were hybridized for 2 h at 20°C with binding buffer containing 40 ng of the biotin-labeled probe (*snail* promotor: 5’-*taa*-*ggg-agt-tgg-cgg*-3’). The positions of the biotin end-labeled oligonucleotides were detected by a chemiluminescent reaction according to the manufacturer’s instructions (Pierce, USA) and visualized by autoradiography.

### Statistical analysis

Derived values were presented as the means ± SD. Dunnett’s *t* test and one-way analysis of variance (ANOVA) were used to assess significant differences among different groups. Statistical significance was determined by the Fisher test. *P* values <0.05 were considered statistically significant.

## Supporting Information

Figure S1 **Effects of CaA on the viability of malignant HaCaT cells.** After cells were exposed to 0.0, 50.0, 100.0, or 200.0 µM of CaA for 24 and 48 h, respectively, their viabilities were measured by use of a cell counting kit-8 assay. The relative ratios of cell viability were determined by comparing growth of cells exposed to no CaA.
(TIF)

Figure S2 **Effects of SB203580 on the cell viability and on the phosphorylation of p38.** (A) The effects of SB203580 on the viability of malignant HaCaT cells. After cells were exposed to 0.0, 5.0, 10.0, 20.0, or 40.0 µM of SB203580 for 24 h, their viabilities were measured by use of a cell counting kit-8 assay. The relative ratios of cell viability were plotted with untreated cells determining the 100% activity level. (B) SB203580 blocked the CaA-induced phosphorylation of p38. After cells were exposed to 0.0, 5.0, 10.0, or 20.0 µM of SB203580 for 6 h, they were exposed to 100.0 µM of CaA for 24 h. Cell lysates were subjected to Western blots with p38 and p-p38 antibodies. GAPDH levels, measured in parallel, served to standardize the values. We chose the concentration of 10.0 µM for further investigation.
(TIF)

Figure S3 **Schematic representation of the** ***snail*** **gene promotor.** The sequence “*gggagttggc”* of the *snail* promoter is similar to *kappaB* DNA elements (*gggynrrrcc*).
(TIF)
