# Supplementary figures and images for: P38/NF-κB/Snail Pathway Is Involved in Caffeic Acid-Induced Inhibition of Cancer Stem Cells-Like Properties and Migratory Capacity in Malignant Human Keratinocyte
Source: PLoS One. 2013 Mar 13;8(3):e58915. doi: 10.1371/journal.pone.0058915 (PMC3596354; doi:10.1371/journal.pone.0058915)

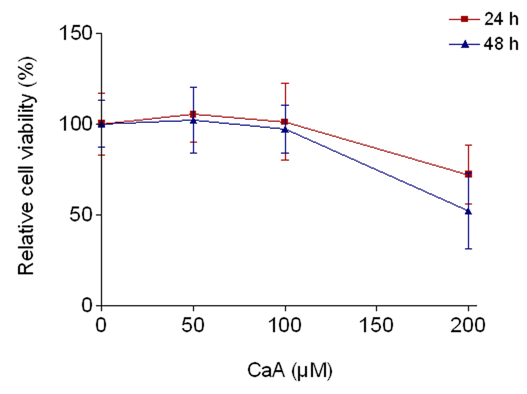

Supplement: Figure S1 — Effects of CaA on the viability of malignant HaCaT cells. After cells were exposed to 0.0, 50.0, 100.0, or 200.0 µM of CaA for 24 and 48 h, respectively, their viabilities were measured by use of a cell counting kit-8 assay. The relative ratios of cell viability were determined by comparing growth of cells exposed to no CaA. (TIF) [file pone.0058915.s001.tif]

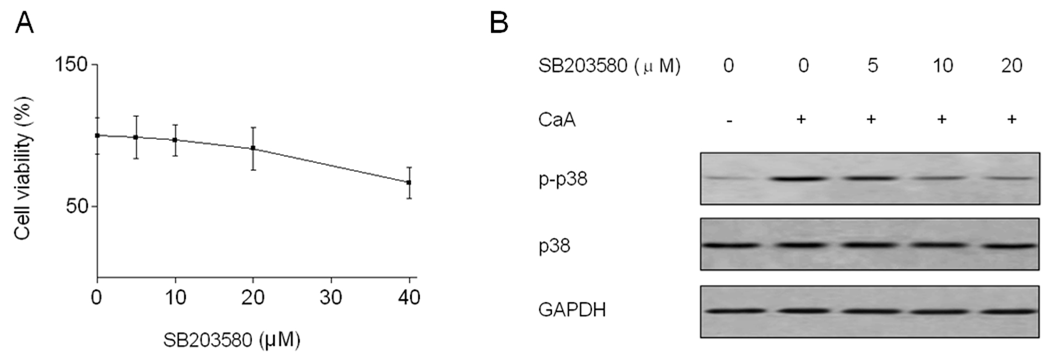

Supplement: Figure S2 — Effects of SB203580 on the cell viability and on the phosphorylation of p38. (A) The effects of SB203580 on the viability of malignant HaCaT cells. After cells were exposed to 0.0, 5.0, 10.0, 20.0, or 40.0 µM of SB203580 for 24 h, their viabilities were measured by use of a cell counting kit-8 assay. The relative ratios of cell viability were plotted with untreated cells determining the 100% activity level. (B) SB203580 blocked the CaA-induced phosphorylation of p38. After cells were exposed to 0.0, 5.0, 10.0, or 20.0 µM of SB203580 for 6 h, they were exposed to 100.0 µM of CaA for 24 h. Cell lysates were subjected to Western blots with p38 and p-p38 antibodies. GAPDH levels, measured in parallel, served to standardize the values. We chose the concentration of 10.0 µM for further investigation. (TIF) [file pone.0058915.s002.tif]

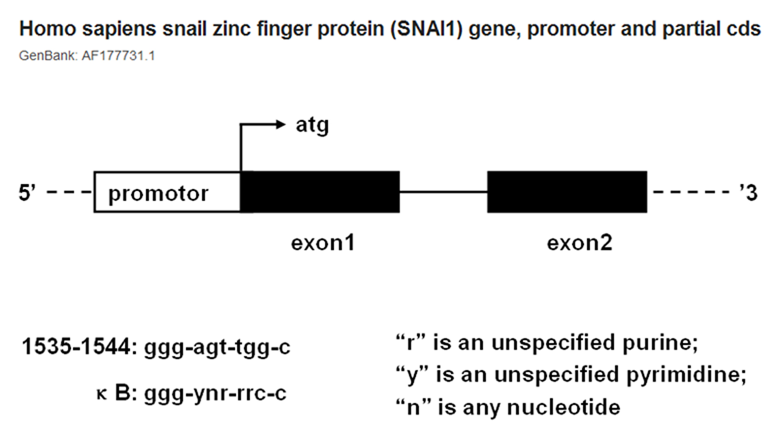

Supplement: Figure S3 — Schematic representation of the snail gene promotor. The sequence “gggagttggc” of the snail promoter is similar to kappaB DNA elements (gggynrrrcc). (TIF) [file pone.0058915.s003.tif]
